# A Synthetic Reporter for Probing Mistranslation in Living Cells

**DOI:** 10.3389/fbioe.2020.00623

**Published:** 2020-06-24

**Authors:** Hao Chen, Carson Ercanbrack, Tony Wang, Qinglei Gan, Chenguang Fan

**Affiliations:** ^1^Cell and Molecular Biology Program, University of Arkansas, Fayetteville, AR, United States; ^2^Department of Chemistry and Biochemistry, University of Arkansas, Fayetteville, AR, United States; ^3^Depratment of Biology, University of Arkansas, Fayetteville, AR, United States

**Keywords:** mistranslation, aminoacyl-tRNA synthetase, acetylation, green fluorescent protein, threonine-tRNA synthetase, editing deficiency

## Abstract

Aminoacyl-tRNA synthetases (AARSs) play key roles in maintaining high fidelity of protein synthesis. They charge cognate tRNAs with corresponding amino acids and hydrolyze mischarged tRNAs by editing mechanisms. Impairment of AARS editing activities can reduce the accuracy of tRNA aminoacylation to produce mischarged tRNAs, which cause mistranslation and cell damages. To evaluate the mistranslation rate of threonine codons in living cells, in this study, we designed a quantitative reporter derived from the green fluorescent protein (GFP). The original GFP has multiple threonine codons which could affect the accuracy of measurement, so we generated a GFP variant containing only one threonine residue to specifically quantify mistranslation at the threonine codon. To validate, we applied this single-threonine GFP reporter to evaluate mistranslation at the threonine codon with mutations or modifications of threonine-tRNA synthetase and compared it with other methods of mistranslation evaluation, which showed that this reporter is reliable and facile to use.

## Introduction

From genetic information stored in the DNA to functional proteins, there are many processes including DNA replication, transcription, and translation. All of these steps can produce mistakes. But the error rates for each step are quite different, ranging from 10^–8^ during DNA replication (Kunkel and Bebenek, 2000) to 10^–4^ during protein synthesis (Ellis and Gallant, 1982). Although there are many mechanisms during translation to maintain high fidelity of protein synthesis, the error rate of translation is still relatively high, which makes protein mistranslation a remarkable research topic in the field of biochemistry (Hoffman et al., 2017b; Schwartz and Pan, 2017; Evans et al., 2018).

Protein mistranslation brings non-cognate amino acids locally into one specific codon or globally replaces one amino acid with another regardless of codons to generate a series of protein variants which can have impaired protein functions or produce aggregation due to protein misfolding (Mohler and Ibba, 2017). Thus, traditionally, protein mistranslation is thought to be harmful or lethal to cells. Indeed, it causes a wide range of human diseases including neurological disorders, developmental disorders, viral infections, and cancers (Schimmel, 2008; Kapur and Ackerman, 2018; Lant et al., 2019; Ou et al., 2019). However, very recently, a number of studies have shown that protein mistranslation is not always detrimental (Pan, 2013; Ribas de Pouplana et al., 2014; Steiner and Ibba, 2019). Misincorporation of methionine can protect cells from reactive oxygen species (Lee et al., 2014). Mischarging of tRNA can mask amino acid starvation to alter stress response signaling (Bullwinkle and Ibba, 2016; Mohler et al., 2017b). Protein mistranslation is also found to be involved in pathogenicity by increasing the antigenic diversity of pathogens to bypass host immune defenses or by promoting phenotypic heterogeneity to increase opportunities to survive under different stress conditions (Li et al., 2011; Miranda et al., 2013; Ackermann, 2015).

To better study protein mistranslation, several methods have been developed to detect or quantify misincorporation of non-cognate amino acids (Ribas de Pouplana et al., 2014; Mohler and Ibba, 2017). The most sensitive and accurate approach is based on mass spectrometry (MS; Mohler et al., 2017a). However, MS cannot be used for *in vivo* studies and depends on high-resolution MS facilities, which could not be easily accessible for all the research groups in this field. To be conveniently used in living cells, several reporters have been derived from the green fluorescent protein (GFP) including eGFP T65V (Nangle et al., 2006), GFP Y66K (Biddle et al., 2015), GFP E222Q (Su et al., 2016), and eGFP D129P variants (Hoffman et al., 2017a). But all these variants have multiple target amino acids at other sites (for example, in eGFP T65V, there are 18 valine residues at other positions), which could confound quantification results. To overcome this issue, in this study, we designed a GFP variant with only one threonine residue to quantify mistranslation of the threonine codon in living cells more precisely. This strategy can be applied to develop different single-target amino acid GFP variant reporters for quantifying mistranslation of specific codons.

## Materials and Methods

### Construction of Super-Folder GFP (sfGFP) Variants

The gene of Thr-free super-folder GFP (sfGFP) was ordered from Integrated DNA Technologies (Coralville, IA, United States). The sequence is in the Supplementary Material. The primers for generating sfGFP variants with a single-threonine residue are listed in Supplementary Table S1. The mutations were made by the Q5 Site-Directed Mutagenesis Kit from New England Biolabs (Ipswich, MA, United States) following the manufacturer’s protocol. The DNA sequences of these variants were confirmed by DNA sequencing (Eurofins Genomics, Louisville, KY, United States). The gene of sfGFP or its variants was transformed into *Escherichia coli* BL21 DE3 cells (New England Biolabs, Ipswich, MA, United States) for expression. Detailed procedures for vector construction are in the Supplementary Material.

### Fluorescence Reading

The strain harboring the plasmid to express wild-type (WT) sfGFP or its variants was inoculated into 2 ml minimal medium (M9 medium and 0.4% glucose) with 100 μg/ml streptomycin and incubated at 37°C overnight, individually. Ten microliters of each overnight culture was added into 190 μl fresh minimal medium with 100 μg/ml streptomycin to OD_600__nm_ ∼ 0.15. The 200 μl mixture was pipetted into a well in a 96-well plate with supplementary 0.1 mM IPTG to induce protein expression. The fluorescence intensity (excitation wavelength 485/20 nm; emission wavelength 528/20 nm) and cell growth (OD_600__nm_) were monitored by a microplate reader with continuous shaking at 37°C for 6 h. Means and standard deviations were calculated by three replicates for initial screening and five replicates for measuring mistranslation rates. Normalized fluorescence was the fluorescence reading divided by the cell culture density (OD_600__nm_) or by the protein yield quantified by ELISA. The mistranslation rate equals 1 minus the rate of non-mistranslation, which is the normalized fluorescence of cells expressing threonine-tRNA synthetase (ThrRS) variants divided by that of cells expressing WT-ThrRS.

### Protein Expression, Purification, and Characterization

For easy purification, a His_6_-tag fused to the WT sfGFP or its variants by PCR used the NEBuilder HiFi DNA Assembly Master Mix Kit. The expression and purification procedures followed the previous protocol (Venkat et al., 2017b). Detailed procedures were in the Supplementary Material. The site-specifically acetylated ThrRS variant was generated by our established genetic incorporation system (Venkat et al., 2017a). Protein concentrations were measured by the Bradford Protein Assay (Bio-Rad, Hercules, CA, United States). Purified proteins were fractionated on a 12% SDS-PAGE gel and visualized by the Bio-Safe Coomassie stain (Bio-Rad). To quantify GFP yields, ELISA was performed with anti-His_6_ tag (Abcam, Cambridge, MA, United States).

### Mass and CD Spectrometry

The LC-MS/MS analyses were performed by Yale University Keck Proteomics Facility following previous protocols (Venkat et al., 2019). The purified ThrRS was digested in gel by trypsin and analyzed by LC-MS/MS on an LTQ Orbitrap XL equipped with a nanoACQUITY UPLC system. The Mascot search algorithm was used to search for the acetyllysine modifications. CD spectrometry was performed by previous protocols (Chen et al., 2019). The CD spectra were recorded on a J-1500 CD spectrometer. Purified proteins were diluted to a concentration of 0.1 mg/ml in 5 mM Tris-HCl pH 7.8, 0.1 M KCl, and scanned from 190 to 250 nm with a 20 nm/min speed. Scanning was performed three times for each sample, and the average was plotted.

## Results

### Developing a Precise Reporter to Evaluate Mistranslation at the Threonine Codon *in vivo*

**FIGURE 1:**
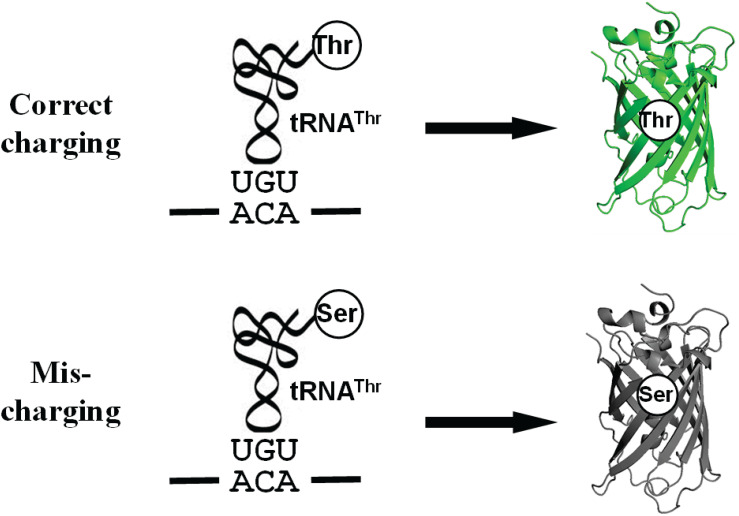
A scheme for the Thr-dependent sfGFP variant. The misincorporation of serine at the threonine codon due to mischarged Ser-tRNA^Thr^ eliminates the fluorescence of sfGFP.

ThrRS uses a zinc ion to discriminate against the valine (without hydroxyl group) at the activation step and utilizes the N-terminal editing domain to hydrolyze mischarged tRNA^Thr^ with serine (with a smaller size) (Sankaranarayanan et al., 2000; Dock-Bregeon et al., 2004). Because ThrRS solves such a unique double-discrimination problem with isosteric amino acids, it has attracted much attention for studying its mistranslation (Dock-Bregeon et al., 2000). However, there is still no facile method to quantify mistranslation of threonine codons in living cells. In this study, we engineered the sfGFP for this purpose. As mentioned, ThrRS can mischarge tRNA^Thr^ with serine. Besides serine, alanyl-tRNA synthetase (AlaRS) can also mischarge tRNA^Thr^ with alanine (Sun et al., 2016). The sfGFP has 18 threonine residues in total. All these threonine codons could be mistranslated as serine or alanine. Thus, we actually observe the overall effect of mistranslation of all these threonine codons on GFP fluorescence. Mistranslation at some sites could increase fluorescence, while that at others could impair fluorescence. So we may underestimate or overestimate the actual mistranslation. To eliminate possible interference from mistranslation at other threonine codons and focus on one specific threonine codon, we aimed to generate an sfGFP variant containing only one threonine residue which is essential for its fluorescence (Figure 1).

**FIGURE 2:**
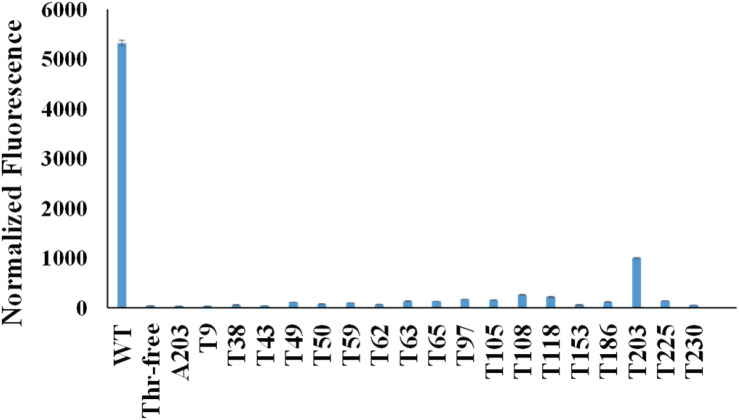
Normalized fluorescence intensities of cells expressing sfGFP variants. Both the fluorescence intensity and the cell culture density of each strain expressing different sfGFP variants were monitored. The fluorescence intensity at 6 h after induction for each strain was normalized by its cell culture density (OD_600__nm_). The fluorescence intensity of the strain harboring the empty vector (pCDF-1b) was used as the baseline which was subtracted from each reading. Mean and standard deviations were calculated based on three replicates.

First, we mutated all the threonine codons in the gene of sfGFP to serine codons because serine is the most common mistranslated amino acid for threonine codons. As expected, this Thr-free sfGFP variant (TF-sfGFP) had no fluorescence (Figure 2). The protein yield of TF-sfGFP was similar to that of WT-sfGFP with the same expression condition (Supplementary Figure S1), indicating that substitution of threonine residues with serine at all 18 sites does not affect GFP expression significantly. We also performed CD spectrometry analyses with both TF-sfGFP and WT-sfGFP to see whether such substitution can affect GFP folding. The result showed that there was no significant difference between them (Supplementary Figure S2). In the next step, we put the threonine codon back to its original position in the gene of TF-sfGFP individually to generate 18 single-threonine sfGFP variants in total. We measured fluorescence intensities for these sfGFP variants (Figure 2). Among these variants, the TF-sfGFP T203 variant restored ∼20% fluorescence with one threonine residue alone. We also noted that protein yields of all these variants were similar to those of WT-sfGFP with the same expression condition (Supplementary Figure S1).

T203 plays a critical role in forming the structure similar to a proton pump for GFP and is well conserved among GFP-like proteins (Ong et al., 2011), which explained the recovery of sfGFP fluorescence. Besides T203, T65 is well known to increase the fluorescence of GFP (Heim et al., 1995). But the TF-sfGFP T65 variant in this study had no significant fluorescence, probably because it is a good enhancer, but not essential for producing fluorescence. Actually, the original amino acid at this site in WT-GFP is serine. Besides serine, AlaRS can also mischarge tRNA^Thr^ with alanine (Sun et al., 2016). To eliminate the possibility that the substitution of threonine with alanine at position 203 can also generate fluorescence, we generated the TF-sfGFP A203 variant. This variant had no fluorescence (Figure 2). Thus, the TF-sfGFP T203 variant can be used as the reporter for quantifying mistranslation of the threonine codon, because (i) mistranslation of the threonine codon to serine or alanine will eliminate the fluorescence of sfGFP; (ii) there is no interference of mistranslation of other threonine codons in the sfGFP; and (iii) the fluorescence intensity depends on the ratio of mistranslated sfGFP and total amounts of sfGFP.

### Testing the Reporter for Mistranslation of Threonine Codons

In the previous quantitative MS study on ThrRS-mediated mistranslation, the ThrRS editing-deficient variant ThrRS C182A was used to evaluate the mistranslation rate at threonine codons (Mohler et al., 2017a). To compare our method with the MS approach, we expressed the TF-sfGFP T203 variant in the strain containing ThrRS C182A. The strain containing WT-ThrRS was used as the control (Table 1). Both the protein yield and cell culture density were decreased in the strain containing ThrRS C182A, which is consistent with the previous study (Ling and Söll, 2010). To address potential effects of *E. coli* cell autofluorescence on GFP fluorescence readings, which could be caused by different cell densities and stress conditions (Mihalcescu et al., 2015; Surre et al., 2018), we used the corresponding stains harboring the same vector but without the reporter gene as controls. Fluorescence readings were subtracted with corresponding backgrounds. Moreover, we compared two approaches to normalize fluorescence intensities, either by cell culture densities from OD_600__nm_ (Supplementary Figure S3) or by protein yields from ELISA. Both normalization approaches had similar results and showed that the mistranslation rate caused by the ThrRS C182A variant is ∼3%, which is consistent with previous quantitative MS studies (Mohler et al., 2017a). As cell culture densities were monitored simultaneously with fluorescence reading, we used this more facile approach for later experiments.

**TABLE 1:** Comparison of two normalization methods for fluorescence intensities.

	**WT-ThrRS**	**ThrRS C182A**
Fluorescence reading	1,25617	97912
sfGFP yield by ELISA (g/L)	0.4020.013	0.3230.016
Normalized fluorescence by ELISA*	3,12414	3,03110
Mistranslation rate by ELISA**	0	3.10.6%
Cell culture density (OD_600__nm_)	0.6680.019	0.5380.013
Normalized fluorescence by OD_600__nm_	1,88011	1,82012
Mistranslation rate by OD_600__nm_	0	3.20.9%

*Normalized fluorescence was fluorescence readings subtracted with corresponding backgrounds of no insert vector control and divided by the sfGFP yield quantified by ELISA or the cell culture density by OD_600__*nm*_ at 6 h after induction. Mean and standard deviations were calculated based on five replicates. Mean and standard deviations for normalized fluorescence were calculated based on values of normalized fluorescence for each sample rather than normalizing averaged raw fluorescence reading by averaged ELISA or OD values. **The mistranslation rate equals 1 minus the rate of non-mistranslation, which is the normalized fluorescence of cells expressing the ThrRS C182A variant divided by that of cells expressing WT-ThrRS.

**FIGURE 3:**
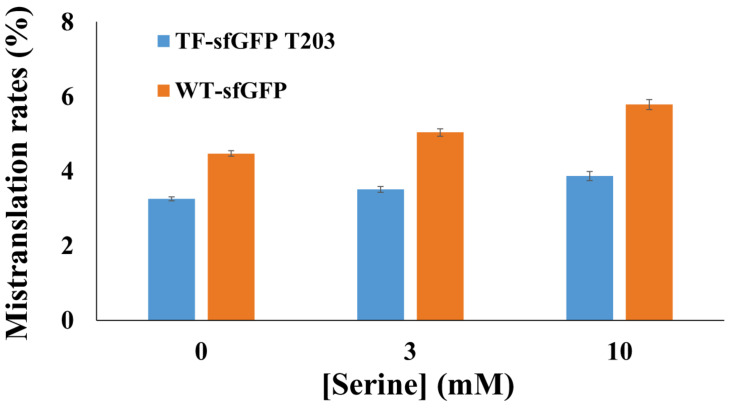
The effect of serine concentrations on sfGFP reporters. Mistranslation rates were calculated with normalized fluorescence by cell culture densities. Mean and standard deviations were calculated based on five replicates.

To compare TF-sfGFP T203 and WT-sfGFP in evaluating mistranslation, we tested the effect of the ThrRS C182A variant on their fluorescence, individually. We also applied a strategy similar to that used in previous mistranslation studies (Lee et al., 2006) and added serine in growth media to force threonine-to-serine mistranslation (Figure 3). With the increase of serine concentrations, both reporters had increased mistranslation rates as expected. WT-sfGFP gave higher mistranslation rates than the single-threonine sfGFP reporter at all conditions, probably because threonine-to-serine substitution at other threonine sites further decreased its fluorescence. In this case, we could overestimate mistranslation rates by using WT-sfGFP as the reporter. Furthermore, the single-threonine sfGFP reporter was less sensitive to serine concentrations than WT-sfGFP, which is another advantage of this reporter.

In our recent study on acetylation of ThrRS, we found that acetylation of K169 in *E. coli* ThrRS can generate Ser-mischarged tRNA^Thr^ *in vitro* (Chen et al., 2019). However, the effect of acetylation of ThrRS on mistranslation of threonine codons in living cells is unknown. So we applied the single-threonine sfGFP reporter to evaluate the impact of acetylation of ThrRS on threonine mistranslation *in vivo*. The site-specifically acetylated *E. coli* ThrRS at K169 (ThrRS-169AcK) was generated by the genetic code expansion strategy in *E. coli* cells and confirmed by LC-MS/MS (Supplementary Figure S4). The TF-sfGFP T203 variant was co-expressed in the strain containing ThrRS 169AcK. Results showed that the mistranslation rate caused by ThrRS acetylation was 4.13 ± 0.06%. K169 is located at the opening of the editing site of ThrRS (Supplementary Figure S5). We proposed that the ThrRS-169AcK variant has an impaired activity to hydrolyze mischarged Ser-tRNA^Thr^ due to steric hindrance from the additional acetyl group. The crystallography study on this acetylated ThrRS variant is ongoing.

## Discussion

Although several GFP variants have been developed to estimate protein mistranslation in living cells (Nangle et al., 2006; Biddle et al., 2015; Su et al., 2016; Hoffman et al., 2017a), they cannot precisely quantify mistranslation rates. Take the eGFP T65V as an example; it was made to evaluate valine-to-threonine mistranslation by an editing-defective ValRS in mammalian cells (Lee et al., 2006). There are 18 other valine residues in the eGFP. When those valine residues are substituted with threonine, the folding or fluorescence properties could also be altered. In this case, the mistranslation rate could be underestimated because valine-to-threonine substitution at other positions can decrease its fluorescence. Actually, authors in that study realized this problem and used a 10-fold free threonine concentration in the medium to force the valine-to-threonine mistranslation at all valine codons. They noticed a decreased mistranslation rate (from 16.7 to 14.4%) and concluded that this number “loosely” reflects the degree of mistranslation in cells (Lee et al., 2006).

In this study, we generated an sfGFP variant with only one threonine codon, so there is no interference from other positions, which makes this strategy unique. This strategy could also be applied in other aminoacyl-tRNA synthetases (AARSs)-mediated mistranslation studies. For the example of ValRS mischarging studies, a Val-free GFP variant could be generated by replacing valine residues with isosteric amino acids such as alanine. By searching for the GFP-like protein data bank (Ong et al., 2011), there is no conserved valine residue in GFP, so it is possible to generate a Val-free GFP variant without eliminating fluorescence. Then the Val-free GFP with T65V substitution could be used for precisely quantifying the mistranslation rate at valine codons.

## Data Availability Statement

All datasets generated for this study are included in the article/Supplementary Material.

## Author Contributions

HC, CE, TW, and QG performed the experiments. HC and CF analyzed the data and wrote the manuscript. All authors contributed to the article and approved the submitted version.

## Conflict of Interest

The authors declare that the research was conducted in the absence of any commercial or financial relationships that could be construed as a potential conflict of interest.
