# Supplementary material for: A Synthetic Reporter for Probing Mistranslation in Living Cells
Source: Front Bioeng Biotechnol. 2020 Jun 24;8:623. doi: 10.3389/fbioe.2020.00623 (PMC7326783; doi:10.3389/fbioe.2020.00623)
Supplement: Supplementary file 1 [file Data_Sheet_1.PDF]

Supplementary Material

A Synthetic Reporter for Probing Mistranslation in Living Cells

Hao Chen[1], Carson Ercanbrack[2], Tony Wang[3], Qinglei Gan[2], Chenguang Fan[1,2*]

[1]Cell and Molecular Biology Program, University of Arkansas, Fayetteville, AR, USA

[2]Department of Chemistry and Biochemistry, University of Arkansas, Fayetteville, AR, USA

[3]Depratment of Biology, University of Arkansas, Fayetteville, AR, USA

*** Correspondence:**
Chenguang Fan
cf021@uark.edu

Materials and Methods

The DNA sequence of Thr-free GFP*

atgagcaagggcgaagaactgttttcgggcgtggtgccgattctggtggaactggatggtgatgtcaatggtcacaaattcagcgtgcgcggcga
aggtgaaggcgatgcaagcaatggtaaactgtcgctgaagtttatttgcagctcgggtaaactgccggttccgtggccgagcctggtcagctcgct
gtcgtatggtgttcagtgtttcagtcgttacccggatcacatgaaacgccacgactttttcaagtccgcgatgccggaaggttatgtccaagaacgta
gcatctcatttaaagatgacggcagctacaaatcgcgcgccgaagtgaaattcgaaggtgattcgctggttaaccgtattgaactgaaaggcatcg
attttaaggaagacggtaatattctgggccataaactggaatataacttcaattcgcacaacgtgtacatcagcgcagataagcagaagaacggtat
caaggctaacttcaagatccgccataatgtggaagatggcagcgttcaactggccgaccactatcagcaaaacagcccgattggtgatggcccg
gtcctgctgccggacaatcattacctgagctcgcagtctgtgctgagtaaagatccgaacgaaaagcgtgaccacatggtcctgctggaattcgtg
agcgcggccggcatctcgcacggtatggacgaactgtataaaggctcataa

* The mutated Ser codons to Thr codons are marked with yellow.

The protein sequence of Thr-free GFP*

MSKGEELFSGVVPILVELDGDVNGHKFSVRGEGEGDASNGKLSLKFICSSGKLPVPWPSLVS
SLSYGVQCFSRYPDHMKRHDFFKSAMPEGYVQERSISFKDDGSYKSRAEVKFEGDSLVNRIE
LKGIDFKEDGNILGHKLEYNFNSHNVYISADKQKNGIKANFKIRHNVEDGSVQLADHYQQN
SPIGDGPVLLPDNHYLSSQSVLSKDPNEKRDHMVLLEFVSAAGISHGMDELYKGS

* The substitutions of Thr with Ser are marked with yellow.

Construction of sfGFP variants. The gene of Thr-free sfGFP was cloned into the *pCDF* vector by PCR reaction and DNA assembly by the NEBuilder® HiFi DNA Assembly Cloning Kit (New England Biolabs, Ipswich, MA, USA). The forward primer is gtttaactttaataaggagatataccatgagcaagggcgaagaactgttttcgg; the reverse primer is cagcggtggcagcagcctaggttaattatgagcctttatacagttcg-tccataccg. The primers for generating sfGFP variants with single Thr residue are listed in Table S1. The mutations were introduced by PCR reactions with the Q5 Site-Directed Mutagenesis Kit (New England Biolabs) following the manufacturer's protocol.

Protein expression and purification. To express individual sfGFP variant, 10 mL overnight culture was transferred into 400 mL of fresh LB medium supplemented with 100 µg/mL streptomycin and grown at 37°C to an absorbance of ~ 0.6 to 0.8 at 600 nm. Then protein expression was induced by the addition of 0.1 mM IPTG. Cells were incubated at 30°C for an additional 4 h and harvested by centrifugation at 3,000 × g for 15 min at 4 °C. The cell paste was suspended in 15 mL of lysis buffer [50 mM Tris (pH 7.8), 300 mM NaCl, 20 mM imidazole, and 1 mM DTT] and broken by sonication. The crude extract was centrifuged at 20,000 × g for 20 min at 4 °C. The supernatant was filtered through a 0.45-µm membrane and loaded onto a column containing 2 mL of Ni-NTA resin (Qiagen, Hilden, Germany) previously equilibrated with 20 mL lysis buffer. The column was then washed with 20 mL of washing buffer [50 mM Tris (pH 7.8), 300 mM NaCl, and 50 mM imidazole]. The His6-tagged protein bound to the column was finally eluted with 3 mL of elution buffer [50 mM Tris (pH 7.8), 300 mM NaCl, and 150 mM imidazole].

Figure S1

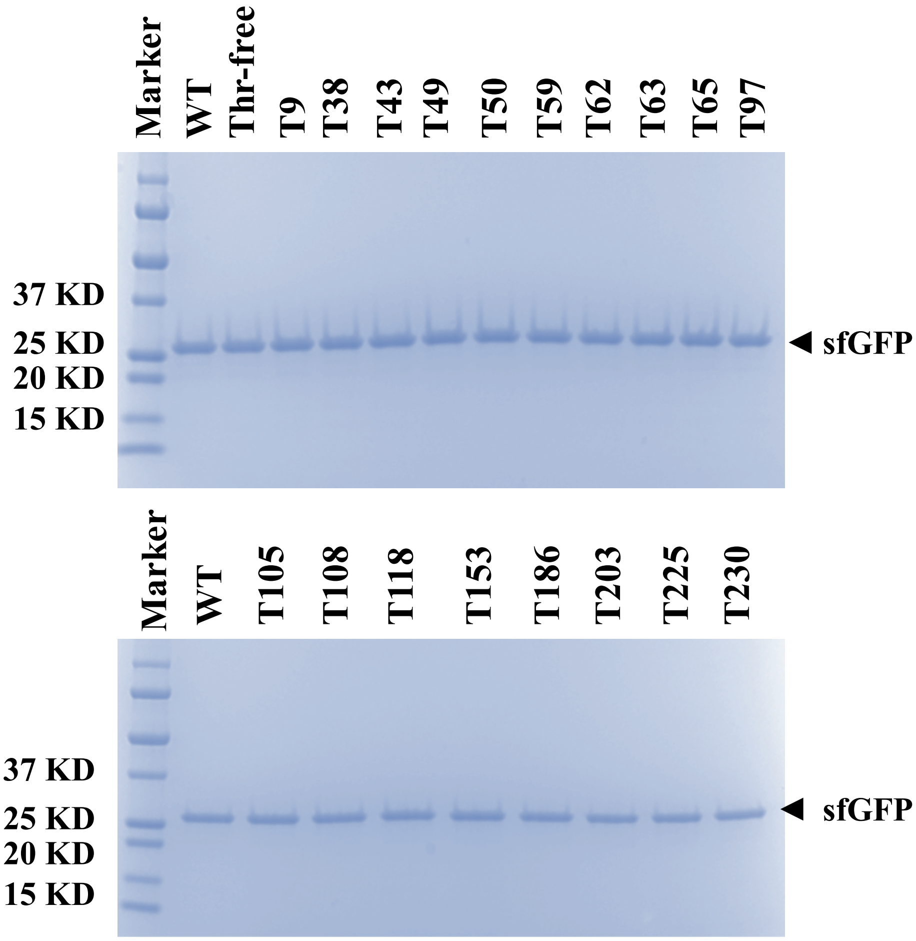

Figure S1. SDS-PAGE of purified sfGFP variants. The sample volume of elution fraction was loaded on the gel for each strain expressing sfGFP variants, respectively.

Figure S2

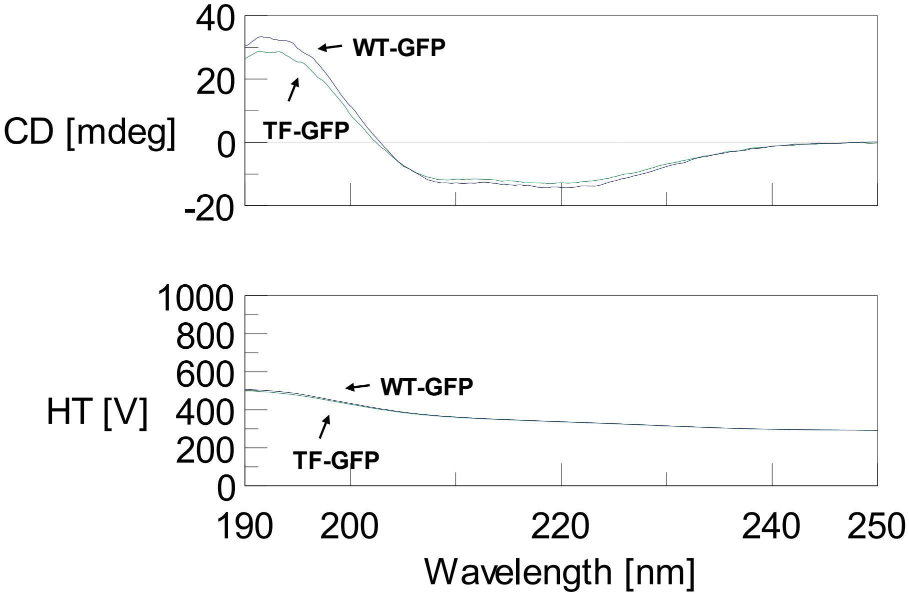

Figure S2. The circular dichroism (CD) spectra of the WT-GFP and TF-GFP. The CD spectra were recorded on a J-1500 CD Spectrometer. Purified proteins were diluted to a concentration of 0.1 mg/ml in 5 mM Tris-HCl pH 7.8, 0.1 M KCl, and scanned from 190 nm to 250 nm with a 20 nm/min speed. Scanning was performed three times for each sample and the average was plotted.

Figure S3

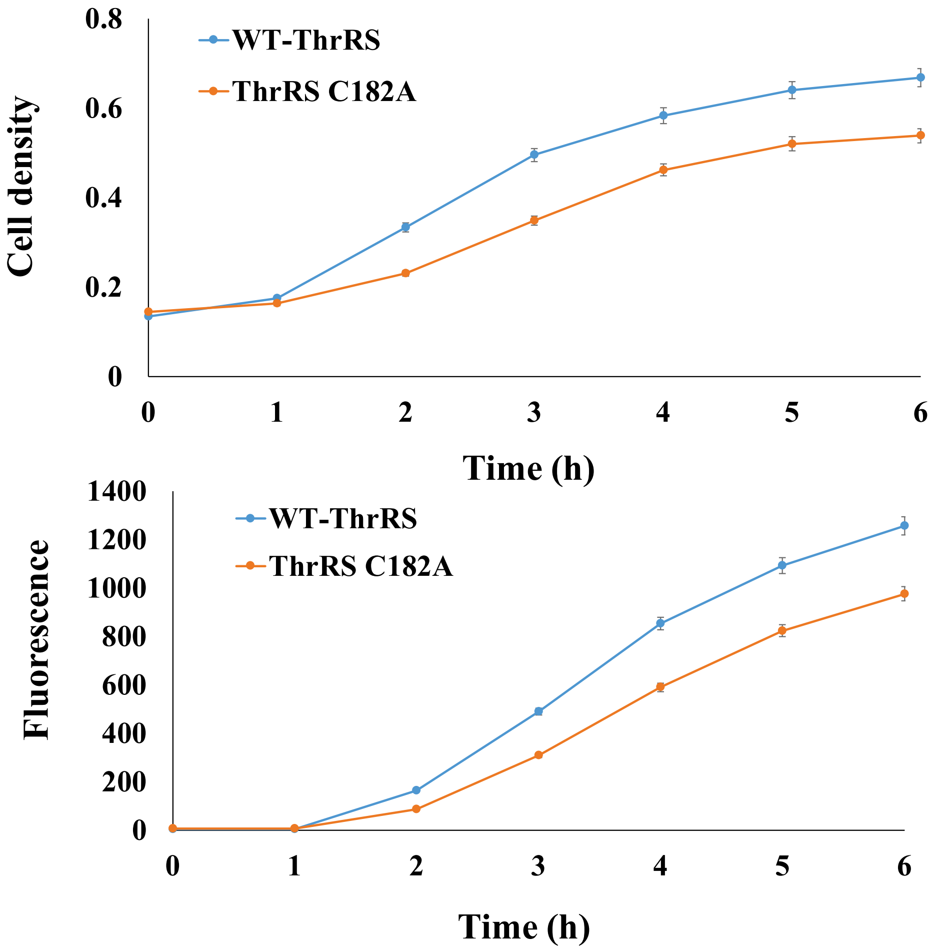

Figure S3. Cell culture densities and fluorescence readings of cells expressing the TF-sfGFP T203 reporter in strains with WT-ThrRS and ThrRS C182 variants, respectively. Mean and standard deviations were calculated based on five replicates.

Figure S4

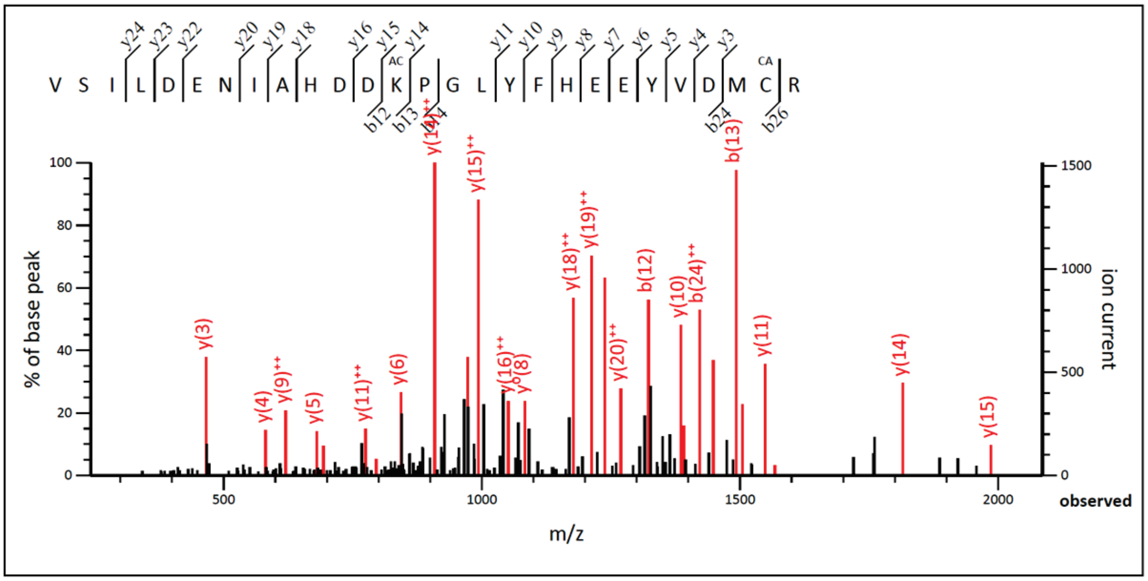

Figure S4. LC-MS/MS analysis of the ThrRS-169AcK variant. The tandem mass spectrum of the peptide (residues 157-183) VSILDENIAHDDKACPGLYFHEEYVDMCR from the purified ThrRS-169AcK variant. K^{AC} denotes AcK (acetyllysine) incorporation. The partial sequence of the peptide containing the AcK can be read from the annotated b or y ion series.

Figure S5

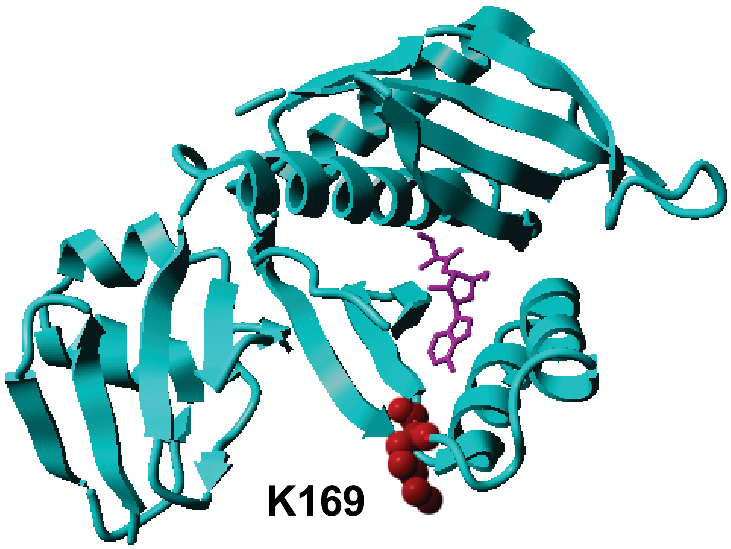

Figure S5. The location of K169 in *E. coli* ThrRS. K169 is shown in the structure of the N-terminal editing site of ThrRS (PDB ID: 1TKG). The nonhydrolyzable analog of seryl-adenylate is in magenta. The lysine residue K169 is marked with red color.

Table S1. The list of primers used for generating sfGFP variants

Variant name	Forward primer	Reverse primer
TFGFP-9T	ctgtttacgggcgtggtgcc	ttcttcgcccttgctcatggtatatc
TFGFP-38T	gatgcaaccaatggtaaactgtcg	gccttcaccttcgccgc
TFGFP-43T	gtaaactgacgctgaagtttatttgc	cattgcttgcatcgccttcac
TFGFP-49T	atttgcacctcgggtaaactgcc	aaacttcagcgacagtttaccattgcttg
TFGFP-50T	atttgcagcacgggtaaactgcc	aaacttcagcgacagtttaccattgcttg
TFGFP-59T	gccgaccctggtcagctc	cacggaaccggcagtttacc
TFGFP-62T	ggtcacctcgctgtcgtatgg	aggctcggccacggaac
TFGFP-63T	ggtcagcacgctgtcgtatgg	aggctcggccacggaac
TFGFP-65T	ggtcagctcgctgacgtatgg	aggctcggccacggaac
TFGFP-97T	gaacgtaccatctcatttaaagatgacgg	ttggacataaccttccggcatc
TFGFP-105T	gacggcacctacaaatcgcgc	atctttaaatgagatgctacgttcttggac
TFGFP-108T	gacggcagctacaaaacgcgc	atctttaaatgagatgctacgttcttggac
TFGFP-118T	gtgatacgctggttaaccgtattgaac	cttcgaatttcacttcggcgcg
TFGFP-153T	tacatcaccgcagataagcagaagaac	cacgttgtgcgaattgaagttatattccag
TFGFP-186T	caaaacaccccgattggtgatgg	ctgatagtggtcggccagttg
TFGFP-203T	ctgagcacgcagtctgtgctgag	gtaatgattgtccggcagcaggac
TFGFP-225T	gaattcgtgaccgcggcc	cagcaggaccatgtggtcac
TFGFP-230T	ggcatcacgcacggtatgg	ggccgcgctcacgaattc
